# A Pair of Sibling Patients With Premature Aging Syndrome of Unknown Etiology

**DOI:** 10.7759/cureus.61300

**Published:** 2024-05-29

**Authors:** Kenji Iwai, Manabu Okawada

**Affiliations:** 1 Pediatrics, Sunrise Japan Hospital Phnom Penh, Phnom Penh, KHM; 2 Pediatric Medicine, Sunrise Japan Hospital Phnom Penh, Phnom Penh, KHM

**Keywords:** premature aging syndrome, hutchinson gilford progeria syndrome, sibling, progeria, short stature

## Abstract

Premature aging syndrome is a rare condition characterized by premature aging and death. The exact pathogenic mechanisms underlying most premature aging syndromes are poorly understood. Here, we describe two sibling cases of premature aging syndrome of unknown etiology, with no identified significant genetic mutation, with the primary symptom of a prematurely aged appearance, and a chief complaint of marked short stature. The first patient was an eight-year-old Cambodian boy born to a third-degree consanguineous marriage. He visited our hospital with the chief complaint of short stature. His development was originally normal until he developed pneumonia when he was three years old. Neither of his parents had any symptoms or family history of similar abnormalities, except for his five-year-old sister, who also has a markedly short stature of 80.4 cm and a low body weight of 8.7 kg. Her face showed distinct macrognathia and relative macrocephaly. The brother’s low-density lipoprotein cholesterol level was high (198 mg/dl), and brain magnetic resonance angiography and carotid ultrasound revealed severe atherosclerotic changes. Whole-exome sequencing results were insignificant for both patients. This case report aims to elucidate the pathogenesis and treatment of progeria. This report indicates the possibility of an unidentified type of premature aging syndrome.

## Introduction

Premature aging syndrome is characterized by premature aging and death. The cause of death varies according to the etiology of the etiology of the exact syndrome. For example, patients with Hutchinson-Gilford progeria syndrome (HGPS) primarily die of atherosclerotic cardiovascular events. Patients with other types of progeria syndromes, such as Werner and Cockayne syndromes, are more susceptible to cancer [1]. In recent years, genetic mutations responsible for premature aging have been identified, including point mutations in the *LMNA* gene for HGPS, an abnormality that can destabilize the nucleus and damage DNA [2], and in the *WRN* gene for Werner syndrome, whose loss of function may cause inhibition of DNA repair, recombination, replication, and transaction [3]. However, the exact pathogenic mechanisms underlying most premature aging syndromes remain poorly understood. Herein, we describe the case of a patient and his sibling with a prematurely aged appearance who presented to our hospital with the chief complaint of marked short stature, who were ultimately diagnosed with premature aging syndrome of unknown etiology, in whom no significant genetic mutation could be identified. This case report shows a type of progeria that has not yet been reported and aims to elucidate the pathogenesis and treatment of progeria.

## Case presentation

Presentation of the sibling cases

**Figure 1:**
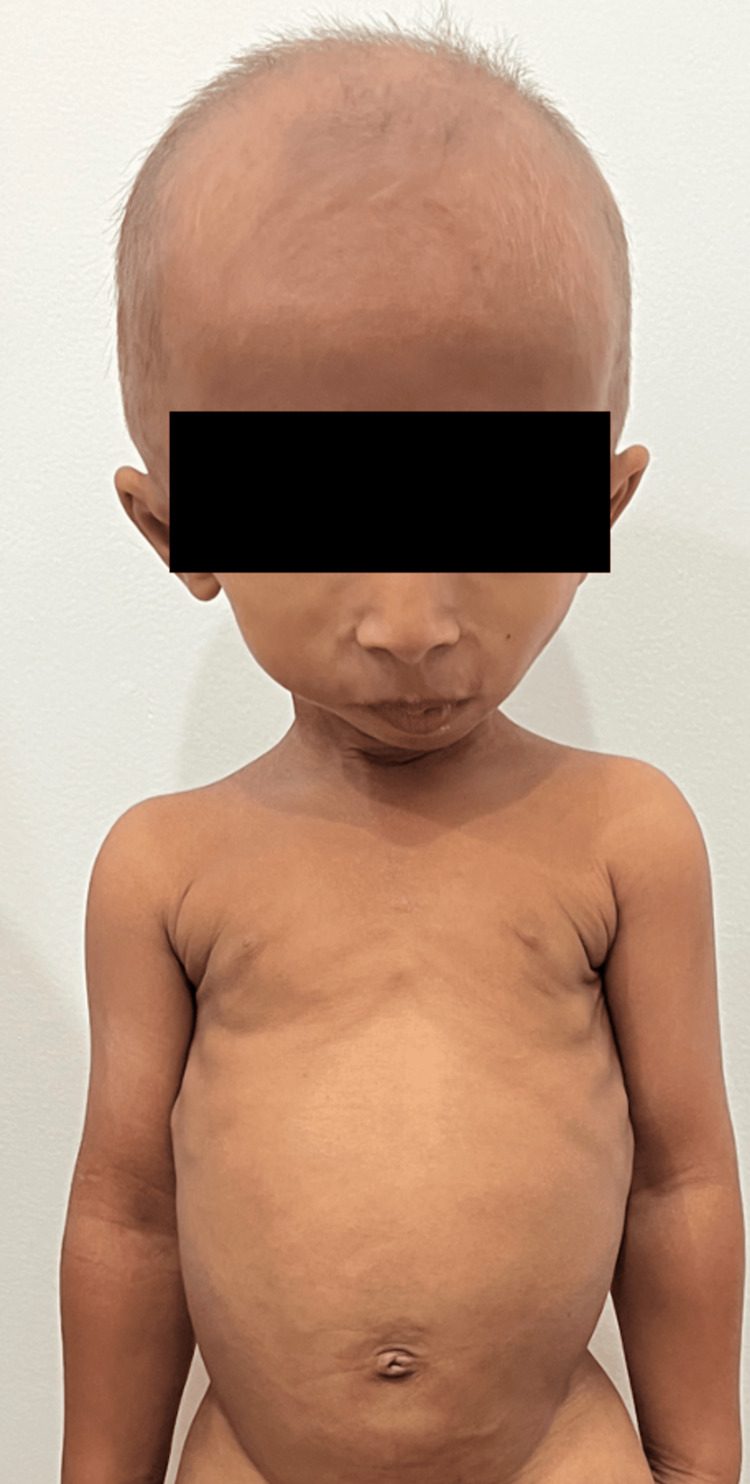
The physical appearance of the patient

The first patient was an eight-year-old Cambodian boy born to a third-degree consanguineous marriage who visited our hospital with the chief complaint of short stature. His development had been normal until he developed pneumonia when he was three years old. However, his parents reported that his physical growth had halted after recovering from pneumonia. The patient was born full-term via standard vaginal delivery with a body weight of approximately 3.0 Kg and had experienced no adverse events during the antenatal period. Neither of his parents had any symptoms or family history of similar kinds of abnormalities, except for his five-year-old sister, who had a markedly short stature of 80.4 cm and a low body weight of 8.7 Kg. On physical examination, the patient’s height was found to be below average (74.5 cm) and his body weight was 7.7 kg. His face was distinctive, showing macrognathia, hair thinning, a relatively long nasal bridge, and relative macrocephaly (Figure 1).

**Figure 2:**
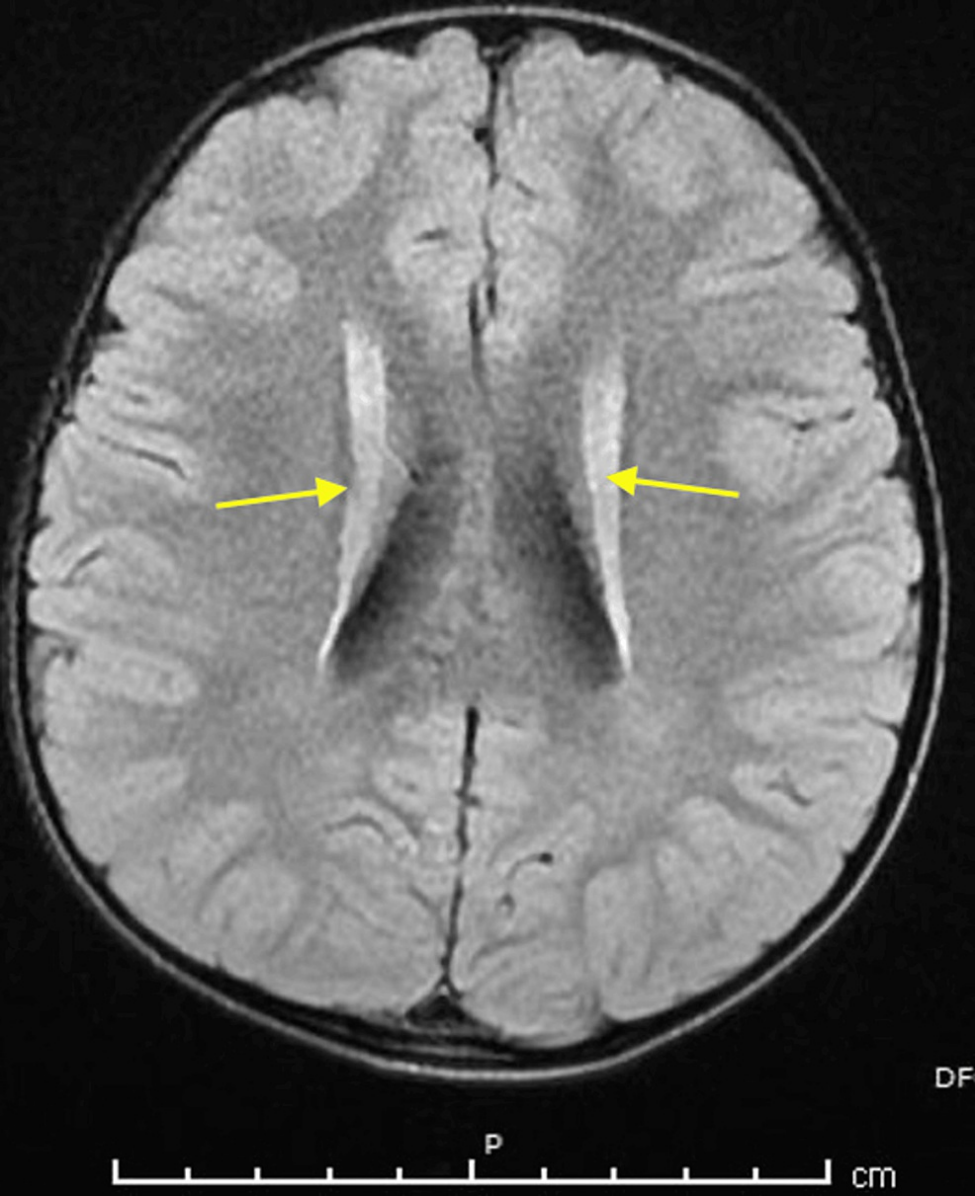
Magnetic resonance imaging of the patient's brain Chronic small vessel ischemia involving the periventricular white matter (Fazekas scale grade 1).

Neuropsychomotor development was appropriate for his age group. Blood tests revealed a high low-density lipoprotein cholesterol level of 198 mg/dl and elevated AST of 60 U/L. Insulin-like growth factor levels were low at 8.30 ng/ml. Brain magnetic resonance imaging (MRI) revealed high signals in the periventricular white matter, suggesting a Fazekas scale of grade 1 (Figure 2).

**Figure 3:**
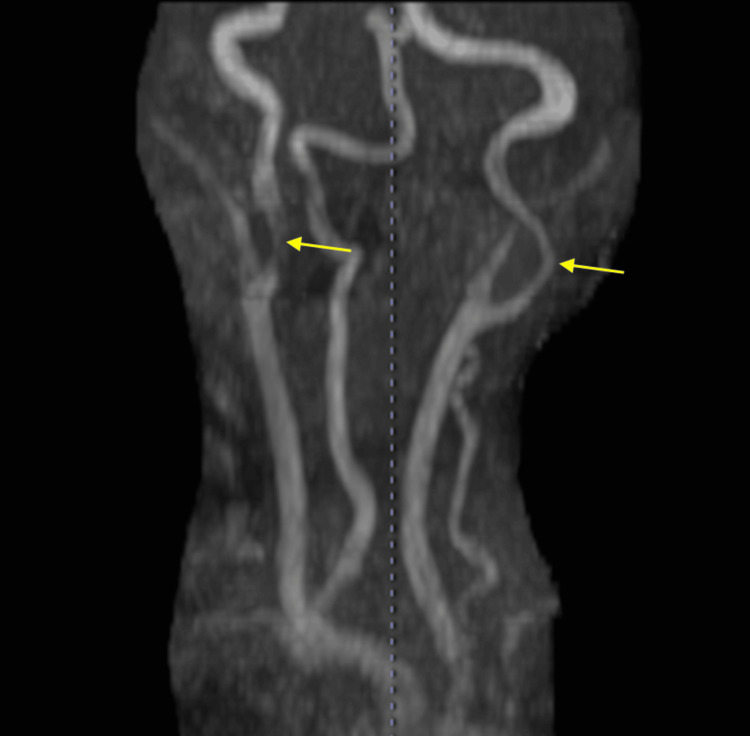
Magnetic resonance angiography of the patient's neck Stenosis of the bilateral internal carotid artery.

Brain magnetic resonance angiography (MRA) revealed significant atherosclerotic changes on both sides of the internal carotid arteries (Figure 3).

Ultrasonography of the carotid arteries revealed a high velocity of 3.8 m/s on the right side and 2.6 m/s on the left side. Currently, he is taking low-dose aspirin (5 mg/kg) and a statin to prevent atherosclerotic cardiovascular events and is undergoing continual follow-up at our hospital. 

**Figure 4:**
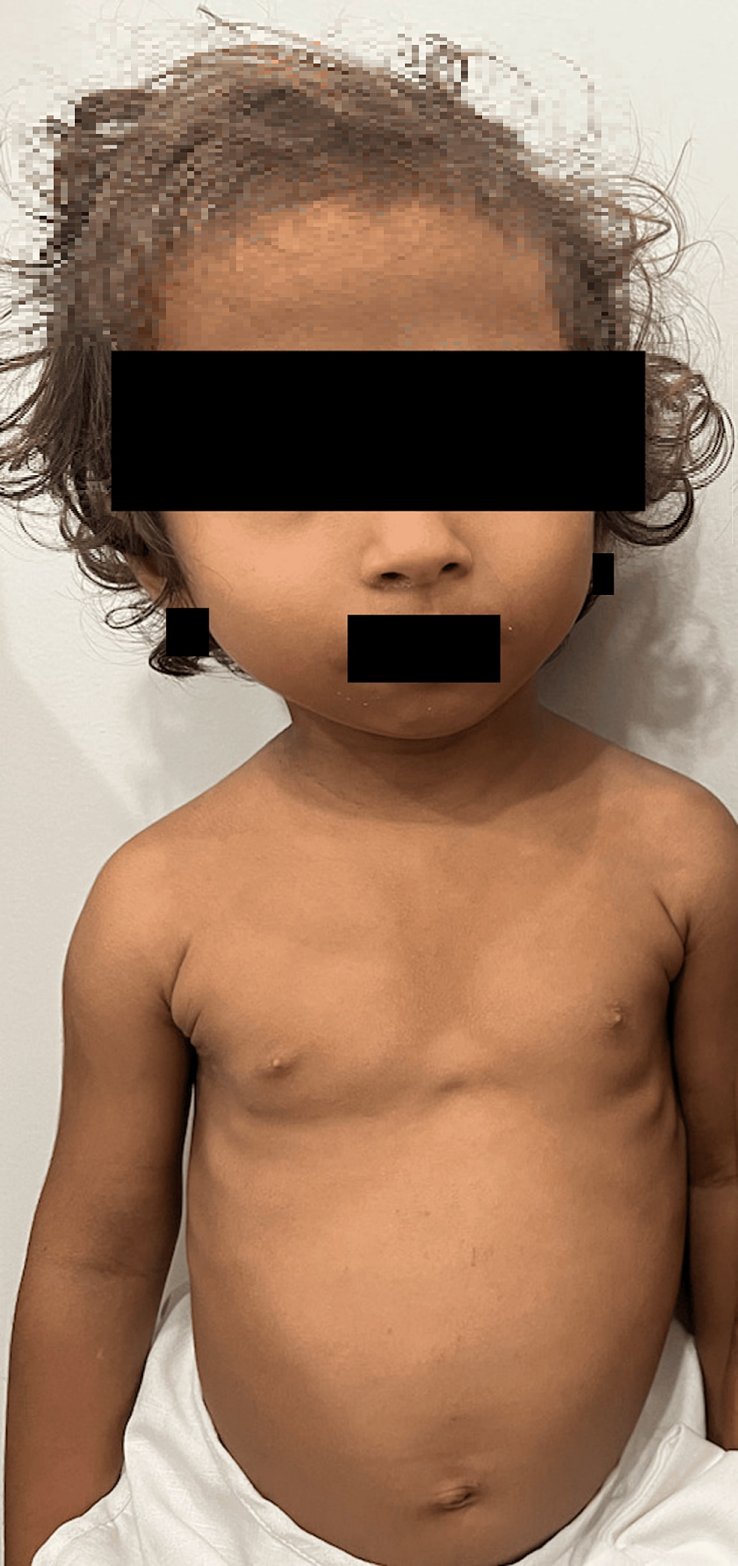
The appearance of the patient's five-year-old sister

The patient’s sister was five years old at the time of her brother’s presentation. She had no significant medical history or history of perinatal events. On physical examination, her height was 80.2 cm and her body weight was 8.5 kg. Her face showed distinctive macrognathia and relative macrocephaly (Figure 4).

**Figure 5:**
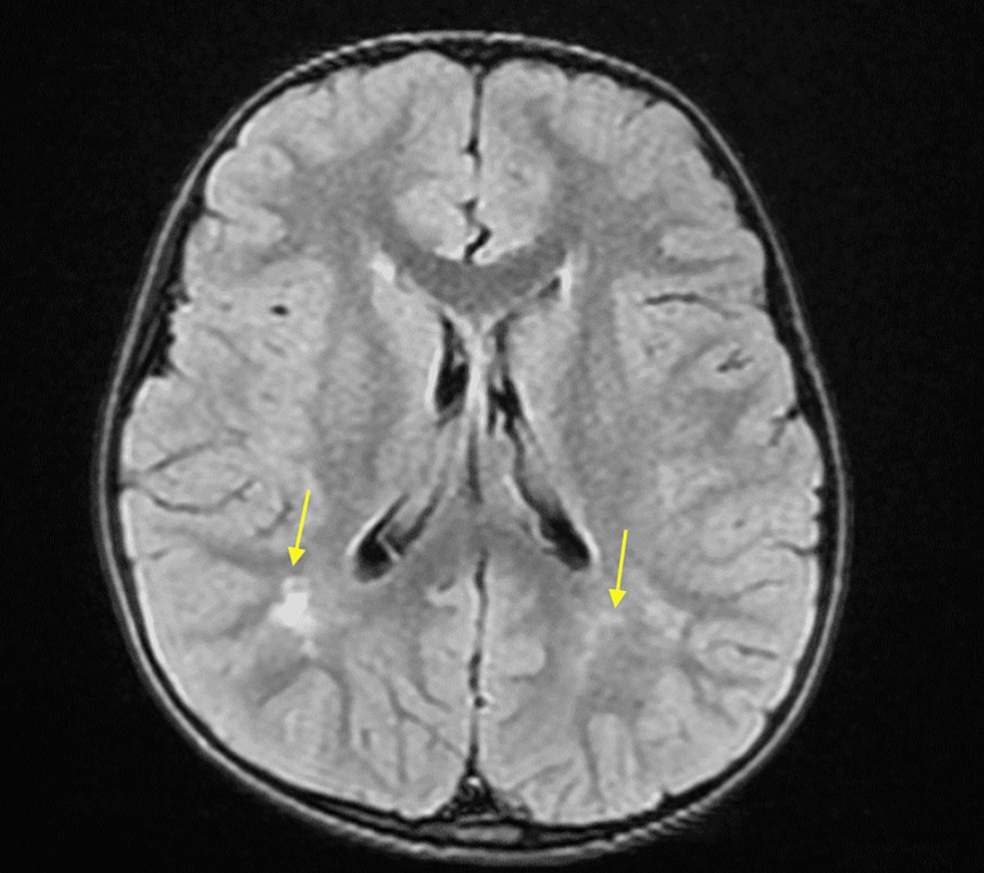
Brain MRI of the patient's five-year-old sister High signals on the bilateral parietal lobe.

Her neuropsychomotor development was appropriate for her age group. Blood tests revealed a high low-density lipoprotein cholesterol level of 116 mg/dl and a slightly elevated aspartate aminotransferase (AST) of 43 U/L. Brain MRI revealed high signals in the bilateral parietal lobe white matter, suggesting chronic ischemic changes (Figure 5), while brain MRA did not show any abnormalities. She continued to be followed up on her general condition and development at our hospital.

Results of whole genome sequencing

To make a clear diagnosis such as HGPS, whole-genome sequencing was performed for the entire family. An autosomal dominant heterozygous variant (c.693C>T. p.lle231=) in platelet-derived growth factor receptor B (*PDGFRB*) and a heterozygous variant (c.1746_1748del, pLeu583del) in the family with sequence similarity 111 member A (*FAM111A*) were identified. These variants were not in the population database for the patient. His mother harbored the same heterozygous variant of *FAM111A*, while his father harbored the heterozygous *PDGFRB* variant. However, both parents were asymptomatic. The patient’s sister harbored the same heterozygous variant in *FAM111A*, as her mother and brother.

## Discussion

Herein, we describe two sibling cases initially suspected to have Hutchinson-Gilford progeria syndrome, but in whom no genetic mutation could be detected, ultimately leading to a diagnosis of progeria of an unknown etiology. Both patients had markedly short stature and low body weight for their age, as well as distinctive facial features. Further, examinations showed evidence of atherosclerotic changes. These features correspond with those of HGPS; however, because no potentially causative genetic mutations were identified, it is possible they had an HGPS-related disease. Genetic analysis of both parents also revealed no abnormalities, although their consanguinity may have made the genetic abnormality slightly more complicated, preventing a typical diagnosis of HGPS. These cases have two clinically important points: both patients had a syndrome that did not fit with the previously reported characteristics of progeria, and this experience suggests the importance of searching for atherosclerotic changes when symptoms that appear to indicate premature aging are observed.

With respect to the first point, physicians must be aware of the possibility of unidentified premature aging syndromes. Initially, we suspected HGPS based on the patient's clinical symptoms and examination results. However, some of the typical symptoms of HGPS, such as skeletal manifestations including contractures, coxa valga, osteolysis, and short clavicles [2], were not observed in these cases. Further, the gene mutation most commonly associated with classic HGPS is a mutation in the *LMNA* gene, comprising a de novo single nucleotide substitution mutation c.1824C > T in exon 11 [4]. This mutation ultimately leads to progerin production, which destabilizes the cell membrane and causes DNA damage [2]. However, in our case, whole-genome sequencing revealed no genetic mutations that could have resulted in symptoms such as those seen in the two cases. In this case, we judged that the gene mutations found in both cases were not responsible for the symptoms, for two reasons. First, despite their mother having the *FAM111A* gene mutation (c.1746_1748del, pLeu583del) and their father having the *PDGFRB* gene mutation(c.693C>T. p.lle231=), neither showed any clinical symptoms. Secondly, the c.693C>T. p.lle231 mutation in *PDGFRB* is a synonymous mutation that does not cause amino acid substitutions and is therefore unlikely to be pathogenic. Although the whole-genome sequencing results could not prove it, autosomal recessive inheritance was suspected here because of the sibling’s findings and the notion of consanguineous marriages, as seen in previous sibling case reports of HGPS [5-6]. Further case series are needed to obtain more accurate information on the disease, including genetic patterns, and to identify more accurate testing methods.

Regarding the second point, although we were unable to make a definitive diagnosis in our case, it was clear that atherosclerotic lesions were present, and that prevention of such lesions was necessary. Although there have been exceptional case reports of HGSP cases with no cardiovascular events until the 30s [7], cardiovascular events have been well-established contributors to early death in HGPS, with patients succumbing on average at approximately 14.5 years old [8,9]. Although internal carotid artery (ICA) stenosis has been reported as one of the causes of cardiovascular events [8], lipid profiles have been reported to be in the normal range for typical HGPS cases [10]. Our two patients had lipid abnormalities, stenosis of the bilateral internal carotid arteries, and chronic ischemic changes in the brain, which are unusual at such a young age. As such, we deemed them to be at risk of early death due to atherosclerotic cardiovascular diseases. This experience suggests that even if a patient’s etiology is unknown and their disease cannot be named, a search for atherosclerotic lesions, lipid profile abnormalities, and prophylactic measures for cardiovascular events should be performed, and follow-up should be performed regularly in patients with progeria symptoms. 

Lonafarnib, a farnesyltransferase inhibitor that prevents progerin accumulation, has been approved for the treatment of HGPS in some countries [11]. One case report revealed the possible effects of a cord blood cell infusion on atherosclerosis and dyslipidemia in HGPS [12]. In addition, a recent report showed that administering everolimus to patients with HGPS resulted in the improvement of diseased cells, and although the diagnosis is uncertain, as in this case, this drug may at some point be applied to our cases as well [13].

## Conclusions

In conclusion, these cases suggest that unidentified types of premature aging syndromes may still exist. In addition, arteriosclerotic lesions are known to be a risk factor for early death in premature aging; as such, when clinicians encounter patients with premature aging without a clear diagnosis, it is necessary to search for arteriosclerotic lesions and to take preventive measures according to severity. However, since the etiology of these cases remains unknown, accumulation of similar cases and long-term follow-up are needed to determine the prognosis.
